# Predicting beneficial effects of atomoxetine and citalopram on response inhibition in Parkinson's disease with clinical and neuroimaging measures

**DOI:** 10.1002/hbm.23087

**Published:** 2016-01-12

**Authors:** Zheng Ye, Charlotte L. Rae, Cristina Nombela, Timothy Ham, Timothy Rittman, Peter Simon Jones, Patricia Vázquez Rodríguez, Ian Coyle‐Gilchrist, Ralf Regenthal, Ellemarije Altena, Charlotte R. Housden, Helen Maxwell, Barbara J. Sahakian, Roger A. Barker, Trevor W. Robbins, James B. Rowe

**Affiliations:** ^1^ Department of Clinical Neurosciences University of Cambridge Cambridge United Kingdom; ^2^ Key Laboratory of Mental Health Institute of Psychology, Chinese Academy of Sciences Beijing China; ^3^ Medical Research Council Cognition and Brain Sciences Unit Cambridge United Kingdom; ^4^ Division of Clinical Pharmacology Rudolf‐Boehm‐Institute of Pharmacology and Toxicology, University of Leipzig Leipzig Germany; ^5^ Department of Psychology University of Cambridge Cambridge United Kingdom; ^6^ Department of Psychiatry University of Cambridge Cambridge United Kingdom; ^7^ Behavioural and Clinical Neuroscience Institute Cambridge United Kingdom

**Keywords:** Parkinson's disease, impulsivity, response inhibition, stratification, noradrenaline, serotonin, machine learning

## Abstract

Recent studies indicate that selective noradrenergic (atomoxetine) and serotonergic (citalopram) reuptake inhibitors may improve response inhibition in selected patients with Parkinson's disease, restoring behavioral performance and brain activity. We reassessed the behavioral efficacy of these drugs in a larger cohort and developed predictive models to identify patient responders. We used a double‐blind randomized three‐way crossover design to investigate stopping efficiency in 34 patients with idiopathic Parkinson's disease after 40 mg atomoxetine, 30 mg citalopram, or placebo. Diffusion‐weighted and functional imaging measured microstructural properties and regional brain activations, respectively. We confirmed that Parkinson's disease impairs response inhibition. Overall, drug effects on response inhibition varied substantially across patients at both behavioral and brain activity levels. We therefore built binary classifiers with leave‐one‐out cross‐validation (LOOCV) to predict patients’ responses in terms of improved stopping efficiency. We identified two optimal models: (1) a “clinical” model that predicted the response of an individual patient with 77–79% accuracy for atomoxetine and citalopram, using clinically available information including age, cognitive status, and levodopa equivalent dose, and a simple diffusion‐weighted imaging scan; and (2) a “mechanistic” model that explained the behavioral response with 85% accuracy for each drug, using drug‐induced changes of brain activations in the striatum and presupplementary motor area from functional imaging. These data support growing evidence for the role of noradrenaline and serotonin in inhibitory control. Although noradrenergic and serotonergic drugs have highly variable effects in patients with Parkinson's disease, the individual patient's response to each drug can be predicted using a pattern of clinical and neuroimaging features. *Hum Brain Mapp 37:1026–1037, 2016*. © **2016 Wiley Periodicals, Inc**.

## INTRODUCTION

There is increasing interest in the development of stratified medicine in neurology and psychiatry, driven by the recognition of patient‐to‐patient heterogeneity in clinical symptoms and treatment responses [Matthews et al., 2014; Schumann et al., 2014; Stephan et al., 2015]. Indeed, the heterogeneity of patients may lead to false‐negative results in clinical trials that rely solely on unselected groups or nonstratified therapies [Sperling et al., 2011; Wardlaw et al., 2014]. The objective of patient stratification is to identify likely responders from nonresponders to maximize the likely efficacy and cost‐effectiveness of a given treatment. Typically it uses demographic and clinical measures but these may be combined with biomarkers such as brain imaging or genotype. In this study, we aimed to build predictive models to identify patient responders in the context of Parkinson's disease, examining the potential of novel noradrenergic and serotonergic therapies for impulsivity.

Previous research has demonstrated that selective noradrenaline (atomoxetine) and serotonin (citalopram) reuptake inhibitors can improve response inhibition in a subgroup of patients with Parkinson's disease. Behavioral performance, brain activity, and/or connectivity were partially restored at either a group level or in a subgroup of patients [Kehagia et al., 2014; Ye et al., 2014, 2015], reinforcing preclinical evidence that these drugs enhance the neural systems for inhibitory control. Here we take a step further to predict the behavioral impact of the drugs in a larger patient cohort, using basic clinical and imaging measures. However, the approach is not limited to these drugs or to Parkinson's disease, but could be implemented in other clinical trials.

In Parkinson's disease, impulsivity is a problem of not only the impulse control disorders present in about 14% of patients [Weintraub et al., 2010]. It occurs even in patients without impulse control disorders [Nombela et al., 2014a; Obeso et al., 2011a]. The inability to stop an action is one of the several dimensions of impulsivity, along with abnormal choices under risk and uncertainty in gambling, delay intolerance, and willingness to respond with insufficient information. This study focuses on response inhibition, in part because of the wealth of animal studies and patient data using response inhibition tasks, and because the potential benefit of atomoxetine has been shown to generalize to other forms of impulsivity [Kehagia et al., 2014].

Response inhibition has been widely studied using the stop‐signal reaction time (SSRT) task in Parkinson's disease and other brain disorders [Cubillo et al., 2014a; Gauggel et al., 2004; Luijten et al., 2013; Obeso et al., 2011a; Verbruggen et al., 2013]. Compared to healthy adults, patients with Parkinson's disease have longer SSRT, reflecting difficulty in cancelling a motor response. The SSRT correlates with ecological and other laboratory measures of impulsivity [Kehagia et al., 2014; Nombela et al., 2014a], with the added advantage that it enables both functional imaging of humans and direct comparison to animal models.

We hypothesized that response inhibition deficits in Parkinson's disease result from loss of noradrenergic and serotonergic projections to the forebrain [Goldstein et al., 2011; Politis et al., 2010], exacerbated by pathological changes in the white matter of frontostriatal circuits that extend from the frontal gyri via the anterior limb of the internal capsule to the basal ganglia [Duncan et al., 2015; Koshimori et al., 2015; Rae et al., 2012; Zheng et al., 2014]. Although dopaminergic dysfunction is a canonical feature of Parkinson's disease and directly linked to impulse control disorders such as pathological gambling [Ray and Strafella, 2013], levodopa withdrawal studies in patients and selective dopaminergic treatments in animal models indicate minimal effects of dopamine on stop‐signal response inhibition [Bari and Robbins, 2013; Obeso et al., 2011b]. In contrast, animal and human studies indicate noradrenergic and serotonergic regulation of response inhibition [Aston‐Jones and Cohen, 2005; Eagle et al., 2008; Robbins, 2007]. For example, atomoxetine and citalopram improve performance on motor inhibition tasks [Chamberlain et al., 2006], reduce premature decisions [Baarendse et al., 2013; Broos et al., 2012], and enhance the associated frontal cortical activations [Chamberlain et al., 2009; Del‐Ben et al., 2005; Macoveanu et al., 2013]. However, the behavioral effects of atomoxetine and citalopram depend on individual differences in the baseline state of noradrenergic and serotonergic systems [Robinson et al., 2008; Ye et al., 2014, 2015] which may be one reason why unstratified studies yield negative results.

This study aimed to identify clinical and imaging features which could enable an accurate prediction of the behavioral effect of atomoxetine (40 mg) and citalopram (30 mg) on response inhibition in Parkinson's disease. We used each drug in addition to standard dopaminergic therapy, in anticipation of a clinical role as adjunctive therapy for nonmotor symptoms rather than substitution of levodopa. We combined the stop‐signal task, pharmacological intervention, brain imaging, and machine learning to develop two models. First, a “clinical” predictive model that indicates whether clinically available information, including demographic measures (e.g., age), clinical measures (e.g., disease severity, cognitive status, levodopa equivalent dose), and a simple baseline diffusion‐weighted imaging measure, is sufficient to predict a patient's response to treatment. We emphasize that such a model is not *in lieu* of a clinical trial, which would require both chronic treatments and clinical outcome measures, but serves to illustrate the potential for patient stratification in future trials. Second, we developed a *post‐hoc* “mechanistic” model that indicates whether behavioral effects can additionally be explained by changes of frontal and striatal activations as measured from functional brain imaging. The advantage of this second model lies in the reinforcement of translational models of impulsivity and Parkinson's disease.

## METHODS

This study was approved by the local research ethics committee and exempted from Clinical Trials status by the UK Medicines and Healthcare products Regulatory Agency.

### Subjects

Thirty‐four patients with idiopathic Parkinson's disease (UK PD Society Brain Bank Diagnostic Criteria) and 42 healthy control subjects with no history of significant neurological or psychiatric disorder participated after providing written informed consent. Thirty‐eight subjects (18 patients with Parkinson's disease and 20 control subjects) contributed to previously published studies [Ye et al., 2014, 2015]. Thirty‐eight different subjects (16 patients with Parkinson's disease and 22 control subjects) were newly recruited and tested following the same research protocol. The two cohorts were combined to increase the ability to effectively fit a predictive model. Three patients from the second cohort had contributed to our previous published studies, and their data have been excluded from the first cohort for the current analysis. All subjects were right‐handed. No patient had dementia (mini mental state examination >26/30), significant current depression, or contraindications to magnetic resonance imaging (MRI), atomoxetine, or citalopram. The patients were tested on their regular antiparkinsonian medications, including levodopa (*N* = 32), dopamine agonists (pramipexole, ropinirole, or rotigotine; *N* = 25), and other medications (amantadine, entacapone, or rasagiline; *N* = 12). Levodopa equivalent dose was calculated according to Tomlinson et al. [2010]. The decision to maintain usual dopaminergic medication was because of the likelihood that either new treatment would be adjunctive to dopaminergic therapy. Table 1 summarizes the demographic and clinical data.

**Table 1:** Mean demographic and clinical measures (standard deviations) and group differences

Measures	Patient^a^	Control	Group difference^b^
Sex ratio (male:female)	21:13	23:19	ns
Age (years)	66.5 (7.0)	66.6 (6.9)	ns
Education (years)	12.5 (5.7)	14.7 (2.8)	ns
Mini mental state examination	28.6 (1.6)	29.3 (1.0)	ns
Duration of symptoms (years)	8.9 (6.3)	–	–
Unified Parkinson's Disease Rating Scale (section III motor subscale)	22.5 (7.8)	–	–
Hoehn and Yahr	2.0 (0.6)	–	–
Schwab and England activities of daily living scale	81.2 (18.5)	–	–
Levodopa actual dose (mg/day)	547.1 (304.0)	–	–
Levodopa equivalent dose (mg/day)	913.7 (522.3)	–	–

a Patients with Parkinson's disease were tested on their regular dopaminergic antiparkinsonian medications.

b P‐values of chi‐squared or unpaired t‐tests as appropriate, corrected for multiple comparisons; ns, not significant.

We screened the patients for impulse control disorders (pathological gambling, hypersexuality, binge eating, and problematic internet use) but did not restrict our subjects to the minority (14%) of patients with impulsive/compulsive disorders, because response inhibition deficits and impulsivity exist in the general population of patients with Parkinson's disease. Indeed, no patient declared symptoms or behaviors indicative of an impulse control disorder. Note that the Questionnaire for Impulsive–Compulsive Disorders in Parkinson's Disease Rating Scale screening tool had not been validated when the study protocol was developed and therefore was not used.

### Experimental Design

The study used a double‐blind randomized placebo‐controlled three‐way crossover design. The patients attended separate sessions at least 6 days apart, including diffusion‐weighted and functional MRI, after 40 mg oral atomoxetine, 30 mg oral citalopram, or an identically overcoated placebo capsule. We used a cross‐over design for its advantages over parallel‐group design in a placebo‐controlled study of this scale. It controls for several potential confounders across sessions, including individual differences in disease severity and progression, severity and distribution of neurotransmitter loss, and dopaminergic drug responsivity, in addition to age, sex, and genetic polymorphisms. Patients were scanned at the same time of the day on each session. The minimal interval of 6 days between sessions minimizes pharmacological carry‐over effects in view of the short half‐life of each drug [Chalon et al., 2003; Rocha et al., 2007]. To reduce practice effects on the drug effect, we randomized the drug order (using permutation within groups of six successive subjects to ensure counterbalancing).

The patients were moved to the scanner for the stop‐signal task and functional imaging 2 h after the drug administration, close to the estimated peak plasma concentration of the drugs. Blood samples were collected immediately before the task and imaging to monitor individual differences in plasma drug concentration that reflects differences in drug absorption and metabolism (see Discussion). The mean plasma drug concentrations were 401.9 ng/mL after atomoxetine (range 31.7–889.0 ng/mL), 40.2 ng/mL after citalopram (6.6–70.3 ng/mL), and 0 ng/mL after placebo. The current range of plasma drug concentration was comparable to that in previous human studies using similar oral dose of atomoxetine [Chamberlain et al., 2009; Kehagia et al., 2014] and citalopram [Hughes et al., 2015]. The control subjects were tested without drug or placebo, to provide normative data on the task and imaging.

The stop‐signal task has been described in detail elsewhere [Ye et al., 2014, 2015]. In brief, it included randomly interleaved 360 Go trials and 80 stop‐signal trials. Go trials had a left/right black arrow (1000 ms) to which subjects responded by pressing left/right buttons with the right hand. On stop‐signal trials, the left/right black arrow turned red, concurrent with a beep, after a short variable delay, and subjects were required to make no response. The stop‐signal delay was adjusted from trial to trial by an online tracking algorithm to maintain 50% successful inhibition. In 40 additional trials, the stop‐signal delay was set to 0 ms (equivalent to NoGo trials).

### Behavioral Data Analysis

We first replicated previous basic findings in behavior and imaging [Ye et al., 2014, 2015] with the combined cohort, and measured drug‐induced changes in SSRT and brain activation for each patient. We then used machine‐learning models to predict individual patients’ changes in SSRT, using the clinical and imaging data, to discriminate patient responders and nonresponders.

Behavioral measures of the stop‐signal task included the SSRT, mean reaction time of correct Go trials, and rate of Go commission errors. The SSRT was estimated using the integration method and adjusted for omission errors [Ye et al., 2014, 2015]. Shorter SSRTs indicate better stopping efficiency. We examined group differences on all behavioral measures using two‐sample *t* tests (PD‐placebo *versus* control) and the effect of each drug on SSRT separately using repeated‐measures ANCOVAs. The ANCOVA had drug (atomoxetine/citalopram *versus* placebo) as a within‐subject factor and controlled for individual differences in age, disease severity (Unified Parkinson's Disease Rating Scale, UPDRS, III‐motor subscale), cognitive status (mini mental state examination), levodopa equivalent dose, and plasma drug concentration.

### MRI Acquisition and Analysis

MRI was acquired in two stages on the same Siemens Trio 3T scanner with a 12‐channel headcoil (Siemens Healthcare, Erlangen, Germany). The first 38 subjects were tested in 2011–2012 and the second 38 subjects were tested in 2012–2013. For all subjects, diffusion‐weighted images were collected along 63 gradient directions (single acquisition, 63 sequential ascending axial slices, 192 × 192 mm^2^ field of view, 2 mm isomorphic resolution) and analyzed with FSL4.1 following a standardized FSL pipeline (http://www.fmrib.ox.ac.uk/fsl). The images were corrected for head movements and eddy currents, and smoothed with a 2.5‐mm Gaussian kernel. Diffusion tensors were linearly fitted to the images. Images of fractional anisotropy and mean diffusivity were computed, adjusted for outlier values, registered to a study‐specific template (i.e., the fractional anisotropy image of a subject that required the least transformation to the fractional anisotropy images of all other subjects), and normalized to the MNI152 space. Mean skeletons were derived and thresholded at a fractional anisotropy of >0.2 to represent the center of the white matter tracts common to all subjects. Values of fractional anisotropy and mean diffusivity were extracted from the skeletonized anterior internal capsule of frontostriatal connections using anatomically defined masks based on the Johns Hopkins University white‐matter atlas. This region was chosen as most relevant to our hypothesis of the role of frontostriatal circuits [Ye et al., 2014, 2015] and in view of the prior evidence of white matter involvement of this region in Parkinson's disease [Duncan et al., 2015; Rae et al., 2012].

Functional images of the first stage used a “silent” echo planar imaging sequence (32 sequential descending axial slices, 2656 ms repetition time, 44 ms echo time, 78° flip angle, 192 × 192 mm^2^ field of view, 3 mm thickness, 0.75 mm gap, and 3 × 3 mm^2^ in‐plane resolution). The silent sequence was used to create a quiet and patient‐friendly environment for a repeated‐measures design to study the stop‐signal task with auditory stimuli. However, the quiet sequence was not optimal for image quality in the midbrain. We therefore switched to a second sequence at the second stage, with other acquisition parameters adjusted accordingly. Functional images of the second stage used a “standard” echo planar imaging sequence (32 sequential descending axial slices, 2000 ms repetition time, 30 ms echo time, 78° flip angle, 192 × 192 mm^2^ field of view, 3 mm thickness, 0.75 mm gap, and 3 × 3 mm^2^ in‐plane resolution). Within each stage all acquisition parameters were kept consistent across subjects and sessions.

All functional images were preprocessed and analyzed with SPM12 (http://www.fil.ion.ucl.ac.uk/spm) following a same pipeline. We controlled potential effects of acquisition sequences by including sequence as a between‐subject factor in the group‐level whole‐brain analysis. The first 11 volumes were discarded to allow magnetization equilibration. The functional images were realigned to the mean functional image, corrected for acquisition time difference, normalized to the MNI space, smoothed with a Gaussian kernel of 6‐mm full‐width half‐maximum (FWHM), and filtered with a 128 s high‐pass filter.

A general linear model was built for each subject to model the stop‐signal trials and Go trials, separately for successful and failed trials. The subject‐level general linear models convolved a design matrix with the canonical hemodynamic response function. Six parameters of head movement (translations and rotations) were integrated into a single parameter, i.e., total displacement [Wilke, 2012], to increase degrees of freedom and statistical power of the subject‐level model. Classical parameter estimation was applied with a one‐lag autoregressive model. We examined group differences on the “successful‐stop > Go” contract (stop‐related activations) using a two‐sample *t* test (voxel‐level *p* < 0.001, cluster‐level *p* < 0.05 family‐wise‐error‐corrected for multiple comparisons).

The whole‐brain analysis was followed by a region‐of‐interest analysis to examine the effect of each drug on the right inferior frontal gyrus (RIFG). The RIFG region was defined as the intersection of the anatomical definition (Automated Anatomical Labelling) and “successful‐stop > Go” contrast in control subjects, to provide anatomically well defined, stop‐related but unbiased regions‐of‐interest for further analysis within patient subjects. For each drug, parameter estimates for “successful‐stop > Go” were extracted and entered into a repeated‐measures ANCOVA controlled for age, disease severity, cognitive status, levodopa equivalent dose, and plasma drug concentration.

### Construction of Predictive Models

The primary objective of this study is to discriminate patient responders and nonresponders (binary classification) against a benchmark of drug‐induced behavioral improvement using a support vector machine (SVM). We conducted the analysis separately for each drug because preclinical evidence suggests different psychopharmacological effects and neural correlates [Robbins, 2007].

Patients were defined as a responder if they showed an SSRT reduction larger than 30% of the magnitude of Parkinson's deficit in SSRT after drug *versus* placebo (the principal benchmark). This definition balanced the size of drug effect and the rate of patients considered as responders. When the benchmark is too high (e.g., complete resolution of Parkinson's deficit), the model may only enable the recognition of a small group of patients who benefit most from the drug (e.g., 18–26% of the patient cohort, see Supporting Information, Fig. S1). When the benchmark is too low (e.g., behavioral improvement of any size), the model will not be able to distinguish a “meaningful” behavioral improvement from noise. In setting the principal benchmark, we note that it was comparable in scale to typical outcomes of chronic dopaminergic treatments of motor symptoms and those of serotonergic/noradrenergic treatments on affective symptoms [Fahn et al., 2004; Wermuth and Group, 1998; Wiles et al., 2012]. Nevertheless, to assess the robustness of our method to variations in the benchmark, we repeated the analysis against a range of alternative benchmarks from 10% to 50% behavioral improvement, on a group scale and on an individual scale (see Results and Supporting Information).

The analysis was implemented using the open‐source LIBSVM toolbox [Chang and Lin, 2011]. The binary classifier used a radial basis function kernel [*K*, Eq. (1)] with two parameters, the cost function *C* (the cost of misclassifying data points) and kernel parameter *γ*,

(1) $$K\left(x_{i}{,\;x}_{j}\right)\;=\;\text{exp}\;\left(-\gamma \|x_{i}{‐\;x}_{j}{\|}^{2}\right),\gamma >\;0$$

We constructed two models for each drug: (1) a clinical predictive model that used demographic (age, sex), clinical (disease severity, cognitive status, levodopa equivalent dose), and/or diffusion‐weighted imaging measures (fractional anisotropy, mean diffusivity) to predict the effect of drug on behavior (e.g., SSRT‐atomoxetine *minus* SSRT‐placebo); and (2) a mechanistic model that explained the behavioral change in terms of activation changes in the RIFG, pre‐SMA, caudate nucleus, and putamen on successful stop‐signal trials (e.g., activation‐atomoxetine *minus* activation‐placebo). These regions were selected because they play crucial roles in response inhibition [Lorenz et al., 2015; Yu et al., 2015; Zhang et al., 2015] and in mediating the beneficial effect of atomoxetine [Ye et al., 2015].

We assessed performance of the two models using LOOCV, given the limited sample size. The LOOCV has a smaller bias than other validation methods (e.g., split sample validation or twofold cross‐validation) in estimating the “true” prediction error in studies with small samples because each observation has an equal chance to be in a training set and a test set [Molinaro et al., 2005]. For each model, we reported mean prediction accuracy across multiple cross‐validations.

### Optimization of Parameters and Features

We optimized the model parameters (*C*, *γ*) using a “grid‐search” algorithm recommended for LIBSVM [Chang and Lin, 2011]. This algorithm searches across exponentially growing sequences of *C* and *γ* (e.g., *C* = 2^−10^, 2^−9.5^,… 2^10^; *γ* = 2^−10^, 2^−9.5^,… 2^10^) to maximize the cross‐validation accuracy of a given model. We also searched for the best set of features as follows. At each iteration, the algorithm searched available features (e.g., *n* available features) for the feature that increased the cross‐validation accuracy most when combined with features that have been selected (e.g., *m* selected feature). The good feature was then moved from the pool of available features to that of selected features (resulting in *n* − 1 available features and *m* + 1 selected features). The algorithm terminated when adding another feature no longer increased the cross‐validation accuracy, or when all features have been selected.

### Statistical Significance of the Optimized Models

We assessed statistical significance of each model using permutation tests (*p* < 0.05 Bonferroni‐corrected for multiple models). At each iteration, the class label (responder/nonresponder) was randomized and new cross‐validation accuracy was calculated. A distribution of cross‐validation accuracy was generated from 5000 randomizations. The *p*‐value was defined as the rate of random models that showed cross‐validation accuracy larger than the real model (Supporting Information, Fig. S2).

## RESULTS

### Behavioral Results

**Figure 1:**
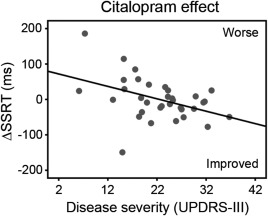
Citalopram reduced the stop‐signal reaction time (SSRT) in patients with more advanced disease (higher UPDRS‐III motor subscale score). ΔSSRT indicates the change in SSRT after citalopram versus placebo.

Compared to control subjects, patients under placebo had longer SSRT (*t* = 2.70, *p* < 0.01), longer Go reaction times (*t* = 3.03, *p* < 0.01), and more Go errors (*t* = 3.19, *p*<0.01). Citalopram, but not atomoxetine, reduced SSRT in patients with more advanced disease (higher UPDRS‐III motor score; see Fig. 1 and Supporting Information, Tables S1 and S2).

### Functional Imaging Results

We first confirmed the previous fMRI findings using the combined cohort (*N* = 76) and replicated the previous result in the subjects who had not participated in our published studies (*N* = 38; see Supporting Information, Figs. S5 and S6).

**Figure 2:**
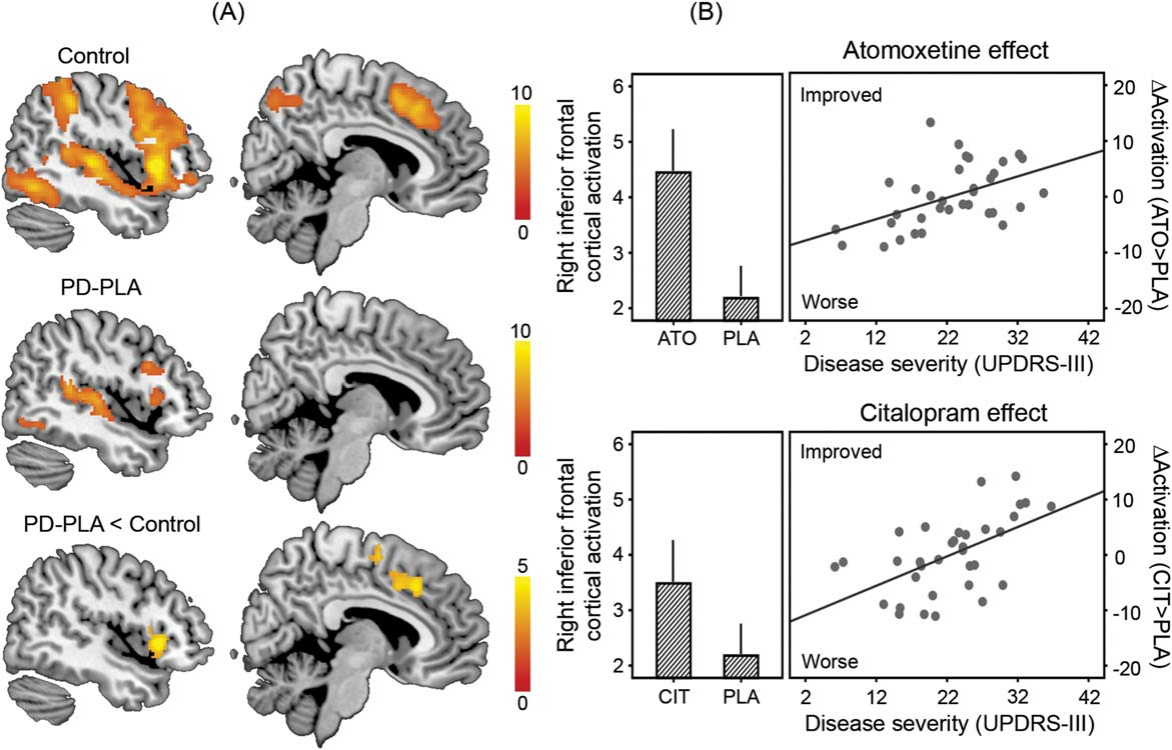
Functional imaging results. (A) Control subjects showed greater activations for successful stop versus go trials (stop‐related brain activations) in the right inferior frontal gyrus and presupplementary motor area. The stop‐related activations were significantly reduced in patients with Parkinson's disease under placebo (PD‐PLA) compared to controls. Statistical parametric maps are overlaid on a representative brain in the MNI space. Colors indicate *t* values of one‐sample or two‐sample *t* tests as appropriate (*p* < 0.05 corrected). (B) In the right inferior frontal gyrus, the stop‐related activation was enhanced after atomoxetine (ATO) and citalopram (CIT) versus placebo (PLA; bar plots), especially in patients with more advanced disease (higher UPDRS‐III motor score; scatter plots). The values of activation are mean parameter estimates adjusted for clinical and demographic covariates. ΔActivation indicates the change in the right inferior frontal cortical activation after drug versus placebo, above the mean improvement in activation. Error bars indicate standard errors.

The whole‐brain analysis revealed group differences on stop‐related brain activation (*p* < 0.05 corrected; see Fig. 2A). Control subjects showed greater activation for successful stop *versus* Go trials in the RIFG (peak in MNI coordinates [44, 20, 4], *t* = 10.93, 1760 voxels) and pre‐SMA ([10, 12, 46], *t* = 7.82, 892 voxels). The stop‐related activations were significantly reduced in patients under placebo compared with controls in the RIFG ([44, 20, −2], *t* = 6.21, 334 voxels) and pre‐SMA ([6, 22, 40], *t* = 4.69, 542 voxels).

The region‐of‐interest analysis revealed the effect of each drug on stop‐related activation (Fig. 2B). Both atomoxetine (*F* = 5.62, *p* < 0.05) and citalopram (*F* = 7.02, *p* < 0.05) enhanced the RIFG activation (main effect of drug), especially in patients with more advanced disease (interaction of drug and disease severity: atomoxetine, *F* = 9.06, *p* = 0.005; citalopram, *F* = 12.42, *p* = 0.001). We present additional analyses of drug effects and results of NoGo trials as Supporting Information.

### Predictive Models for Atomoxetine and Citalopram

**Figure 3:**
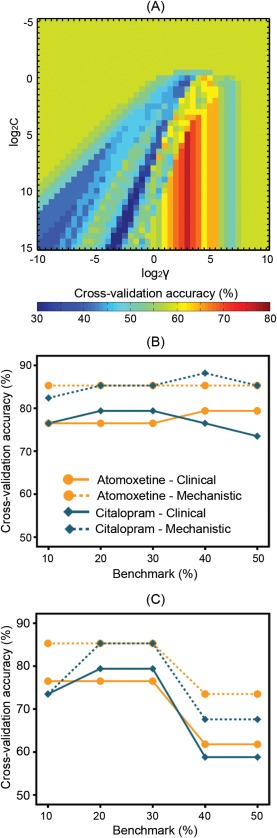
The clinical predictive model and mechanistic model were constructed separately for atomoxetine and citalopram, against the principal benchmark of 30% behavioral improvement. (A) The model parameters were optimized using a “grid‐search” algorithm, which searches across exponentially growing sequences of *C* and *γ* to maximize the cross‐validation accuracy of a given model. The illustrated example used data from the clinical model of atomoxetine response. Colors indicate cross‐validation accuracy values. (B) Cross‐validation accuracy of the models that were optimized for the principal benchmark and for alternative benchmarks (e.g., 10–50% behavioral improvement, see Table 2 and Supporting Information, Tables S3 and S4 for details). (C) Robustness of the optimal models for the principal benchmark was measured as cross‐validation accuracy of the models when tested against alternative benchmarks.

Having confirmed previous findings in behavior and imaging, we then constructed a “clinical” predictive model and a “mechanistic” model for each drug against the principal benchmark of 30% behavioral improvement (*p* < 0.05 corrected; see Table 2), using the “grid‐search” algorithm (Fig. 3A).

**Table 2:** Optimal clinical predictive and mechanistic models against the benchmark of 30% behavioral improvement

Model type	Drug	Optimal features	(*C*, *γ*)	Accuracy	Significance^a^
Clinical	Atomoxetine	L mean diffusivity,^b^ levodopa equivalent dose, R fractional anisotropy, L fractional anisotropy	(2^5^, 2^3^)	76.5%	*p* < 0.05
Clinical	Citalopram	R fractional anisotropy, age, R mean diffusivity, MMSE	(2^10^, 2^0.5^)	79.4%	*p* < 0.05
Mechanistic	Atomoxetine	R caudate nucleus, L caudate nucleus, R pre‐SMA	(1, 2^7^)	85.3%	*p* < 0.05
Mechanistic	Citalopram	L caudate nucleus, R putamen, R pre‐SMA	(2^6^, 2^2^)	85.3%	*p* < 0.05

a Statistical significance measured as p‐values from permutation tests (5000 randomizations, *p* < 0.05 corrected for multiple comparisons).

b Values of fractional anisotropy and mean diffusivity were extracted from the anterior internal capsule. L, left; R, right; pre‐SMA, presupplementary motor area; MMSE, mimi mental state examination.

Using the clinical predictive model, a 30% behavioral improvement after atomoxetine was best predicted by the combination of diffusion‐weighted metrics of the anterior internal capsule and levodopa equivalent dose. For citalopram, the best predictive features included the fractional anisotropy and mean diffusivity of the right anterior internal capsule, age, and cognitive status measured by the mini mental state examination.

In the mechanistic models using *post‐hoc* brain activations, the best predictive features against the principal benchmark were the drug‐induced changes of activation in the bilateral caudate nuclei and right pre‐SMA for atomoxetine, and the activation changes in the left caudate nucleus, right putamen, and right pre‐SMA for citalopram.

The models were similarly accurate to predict individual patients’ responses to the drug against a range of alternative benchmarks (e.g., 10–50% behavioral improvement, see Fig. 3B and Supporting Information, Tables S3 and S4). The optimal models for the principal benchmark were also robust as tested against other benchmarks (Fig. 3C). The sensitivity and specificity of the models are presented in the form of receiver operating characteristic curves as shown in Supporting Information, Figure S7.

## DISCUSSION

Response inhibition is an aspect of impulsivity that is impaired in Parkinson's disease but it is not usually improved by levodopa or dopamine agonists. The limited effect of selective dopaminergic drugs on response inhibition led us to investigate noradrenergic and serotonergic agents as potential adjunctive treatments to regular antiparkinsonian medications. We confirmed the beneficial effect of atomoxetine and citalopram on response inhibition in subgroups of patients with Parkinson's disease, rather than the whole group, and confirmed that the change in behavior (after citalopram, see Fig. 1) and brain activation (both drugs, see Fig. 2B) correlated with the UPDRS measure of disease severity. Note, a groupwise effect of atomoxetine has been observed on stopping accuracy and other behavioral indices of impulsivity in an independent cohort of patients with Parkinson's disease [Kehagia et al., 2014]. In this study, the groupwise effect of the drugs (on brain activation, see the bar plots of Fig. 2B) should not be interpreted in isolation because it showed a significant interaction with the disease severity covariate (see the scatter plots of Fig. 2B). However, such correlations are not sufficient for prospective trialists or clinicians. Greater interest lies in the ability to predict individual patients’ responses to these drugs and stratify interventions accordingly.

We therefore developed a clinical predictive model, which identified potential responders with 77–79% accuracy using a binary classifier and simple measures available before treatment (e.g., age, cognitive status, levodopa equivalent dose, and fractional anisotropy or mean diffusivity measures taken from diffusion‐weighted images; see Table 2 and Supporting Information, Table S3). The principles and methods we used in this model are of direct relevance to the design of future phase II/III clinical trials in heterogeneous populations such as people with Parkinson's disease.

Despite its correlation with the behavioral effect of citalopram, the UPDRS measure was not a consistent feature of the clinical model, possibly because the variance it expresses was partially captured by the diffusion‐weighted imaging measures [Rae et al., 2012]. Although the effects of both drugs are associated with common frontostriatal circuits [Chamberlain et al., 2009; Del‐Ben et al., 2005; Macoveanu et al., 2013], we suggest that the distinct clinical models for atomoxotine and citalopram reflect different roles of noradrenaline and serotonin in inhibitory control [Eagle et al., 2008; Robbins, 2007]. In addition to the clinical, demographic, and neuroanatomical factors we have examined, genetic variations of neurotransmitter transporters or microtubule‐associated protein tau may also play a role in treatment response [Dorszewska et al., 2013; Nombela et al., 2014b; Whelan et al., 2012; Williams‐Gray et al., 2013].

We also present a *post‐hoc* mechanistic model which explained the behavioral effect with 85–88% accuracy in terms of drug‐induced changes in regional brain activation, either alone (see the “mechanistic” models in Table 2 and Supporting Information, Table S4) or in conjunction with clinical measures (see the “mixed” model in Supporting Information, Table S7). Whereas the clinical models for atomoxetine and citalopram are clearly different, the mechanistic models are convergent on the role of the striatum bilaterally along with the pre‐SMA and RIFG. A parsimonious explanation of this convergence is that the frontostriatal circuit integrates several pharmacological processes, and expresses a final common pathway for potential moderators of impulsivity in Parkinson's disease. In previous research [Ye et al., 2015], we included the frontostriatal functional connectivity, in addition to the RIFG activation, in a simple regression model to understand the behavioral improvement after atomoxetine. With a view to a more easily usable model, we did not include such a functional connectivity measure which requires more sophisticated analysis (e.g., pharmacophysiological interactions) before building the classifier.

The effect of atomoxetine and citalopram is unlikely to be mediated by directly increasing cortical dopamine [Bymaster et al., 2002a, 2002b). But an indirect contribution of moderated dopamine neurotransmission is possible, given the significance of the levodopa equivalent dose in the clinical models. Candidate mechanisms for an indirect effect of dopamine include postsynaptic interactions between monoaminergic receptors [Albizu et al., 2011] and reciprocal inhibitory interactions between monoaminergic neurons in the midbrain [Guiard et al., 2008].

We also present clinical and mechanistic models optimized with different feature inputs (see supplemental results). These additional results showed the role and relative importance of each feature (e.g., whether prediction accuracy changed when a particular feature was added into or removed from the model). For example, the inclusion of plasma drug concentration improved prediction accuracy of the clinical model for atomoxetine response (Supporting Information, Table S8), highlighting the importance of measuring individual differences in drug absorption (e.g., transit time and absorption in the gut which is affected by additional autonomic deficits of idiopathic Parkinson's disease) and metabolism (e.g., activity of the cytochrome CYP enzyme family which plays a crucial role in the metabolism of both atomoxetine and citalopram). Like other recent psychopharmacological studies of dopamine, serotonin, and noradrenaline, we did not include measures of receptor occupancy using positron emission tomography [Chowdhury et al., 2013; Costa et al., 2013; van der Schaaf et al., 2013], but rather adopted a simpler approach to incorporate individual differences in plasma drug levels. The plasma drug concentration was measured 2 h after the drug administration and immediately preceding the stop‐signal task and functional imaging, and used as a proxy for the next 20 min of functional imaging. This single measure was sufficient to capture some of the individual difference, although it will not match the peak concentration of each patient (but it was close to the maximal plasma concentration of both drugs in the population, see [Chalon et al., 2003; Rocha et al., 2007]).

## LIMITATIONS

This study has potential limitations. We included patients without clinically evident impulse control disorders. However, 86% of patients with Parkinson's disease do not have impulse control disorders and yet manifest a diverse array of impulsive behaviors on laboratory‐based neuropsychological tests and questionnaires regarding everyday behaviors. Nevertheless, further studies would be helpful to assess the use of atomoxetine and citalopram in the important minority of patients with impulse control disorders for whom reductions in dopaminergic medication may be poorly tolerated.

Another limitation is the extent to which effects on the stop‐signal task generalize to other complex impulsive behaviors or clinically significant nonmotor symptoms. The SSRT is not synonymous with impulsivity but it is a well‐established tool to study inhibitory control systems across many neuropsychiatric disorders, providing a homolog of animal paradigms. In Parkinson's disease, it relates to ecological and other laboratory tests of impulsivity in humans [Kehagia et al., 2014; Nombela et al., 2014a). Stopping efficiency is of course not equivalent to an assessment of the quality of life, activities of daily living, or “everyday impulsivity.” This study was exempted from clinical trials status, as its focus was on the drugs’ effects on neurocognitive systems, and the 30% improvement on stopping efficiency is not a clinical index of improvement on quality of life. However, we chose our definition of the benchmark for responders so as to be comparable in scale to typical outcomes of chronic dopaminergic treatments of motor symptoms and serotonergic/noradrenergic treatments of affective symptoms. For example, dopaminergic treatments typically reduce UPDRS measures of disease severity by 30–40% [Fahn et al., 2004; Wermuth and Group, 1998], while treatments with selective serotonin or noradrenaline reuptake inhibitors typically reduce depression symptom scores on the Beck Depression Inventory by 35% [Wiles et al., 2012]. Nevertheless, further studies directed toward clinical utility will have to incorporate clinical outcome measures.

We investigated the behavioral efficacy of single‐dose atomoxetine and citalopram challenges. The acute and chronic effects of citalopram are comparable in the context of inhibitory control and reinforcement learning [Danet et al., 2012], although not in depression [for a discussion, see Ye et al., 2014]. Our use of single‐dose atomoxetine is in accordance with previous studies in boys with attention deficit/hyperactivity disorder [Cubillo et al., 2014a, 2014b). Given the observed efficacy of acute doses, we propose long‐term treatment studies using an appropriate stratification strategy to increase their power.

We built the predictive models on a relatively small sample of patients with Parkinson's disease, in part due to the resources required for multimodal brain imaging that was motivated by the need to cross‐validate to preclinical models of inhibitory frontostriatal systems. Given the sample size, we used LOOCV to estimate prediction error, as this approach has been shown to be more accurate than other validation approaches (e.g., a split‐sample approach) for small samples [Molinaro et al., 2005]. Nevertheless, studies with larger patient samples are needed to for further verifying the predictive model. While large and late‐stage clinical trials may use an alternative validation method such as *a priori* split‐sampling, other early stage trials could benefit from the imaging‐supported machine‐learning method we present.

One might argue that it would be more cost efficient to simply try patients in clinic on the target drug. However, the efficacy of the drugs would need to have been shown in clinical trials, which are liable to be negative if the wide individual variability in treatment response is not recognized, or pretreatment information is not used to properly stratify patients. Our inclusion of diffusion‐weighted imaging measures in the clinical model could also be challenged, as not all patients undergo brain imaging. However, the diffusion‐weighted measure we propose is basic and suitable for automation from most clinical 3 T MRI scanners (i.e., a simple measure in one region of interest, not a complicated tract‐based index). Moreover, the cost of brain imaging can be considered against the cost of the failure of unstratified clinical trials, the annual cost of ineffective treatments (e.g., current local cost of a National Health Service MRI equates to 2 months treatment of 40 mg atomoxetine), and the risk of harm from treating those in whom a negative response could be predicted.

## CONCLUSION

In conclusion, we have confirmed the potential beneficial effect of atomoxetine and citalopram on response inhibition in selected subgroups of patients with Parkinson's disease, building on a large body of comparative studies. We found that a simple classifier can identify potential responders with high accuracy, while recognizing the lack of consensus on the threshold for response and the important role of clinical outcome measures in chronic treatment trials. The classification method is applicable not only to Parkinson's disease, but also more widely in the translation from preclinical studies to experimental medicine and further toward stratified clinical trials, which is especially relevant to drug development and early stage trials.

## Supporting information

Supporting Information
