# Supplementary material for: Predicting beneficial effects of atomoxetine and citalopram on response inhibition in Parkinson's disease with clinical and neuroimaging measures
Source: Hum Brain Mapp. 2016 Jan 12;37(3):1026–37. doi: 10.1002/hbm.23087 (PMC4819701; doi:10.1002/hbm.23087)
Supplement: Supplementary file 1 — Supporting Information [file HBM-37-1026-s001.doc]

**Supplemental Materials**

Selection of the benchmark

The benchmark was the minimum SSRT reduction that was considered as a ‘meaningful’ behavioral improvement after drug *versus* placebo. For the main analysis, we defined the benchmark as a percentage of the magnitude of the deficit caused by Parkinson’s disease, in SSRT (see Figure S1A). The benchmark can alternatively be defined on an individual scale, for example, as the percentage of individual SSRT under placebo (i.e. SSRT baseline, see Figure S1B). We therefore additionally optimized the clinical predictive and *post-hoc* mechanistic models against a range of different benchmarks (see Table S3-7).

Figure S1: The rate of patients considered as responders, as a function of benchmark selection. The benchmark was defined either (A) as the percentage of the magnitude of Parkinson’s deficit in SSRT, or (B) as the percentage of individual SSRT baseline. The dotted line indicates the principal benchmark of 30% behavioural improvement.


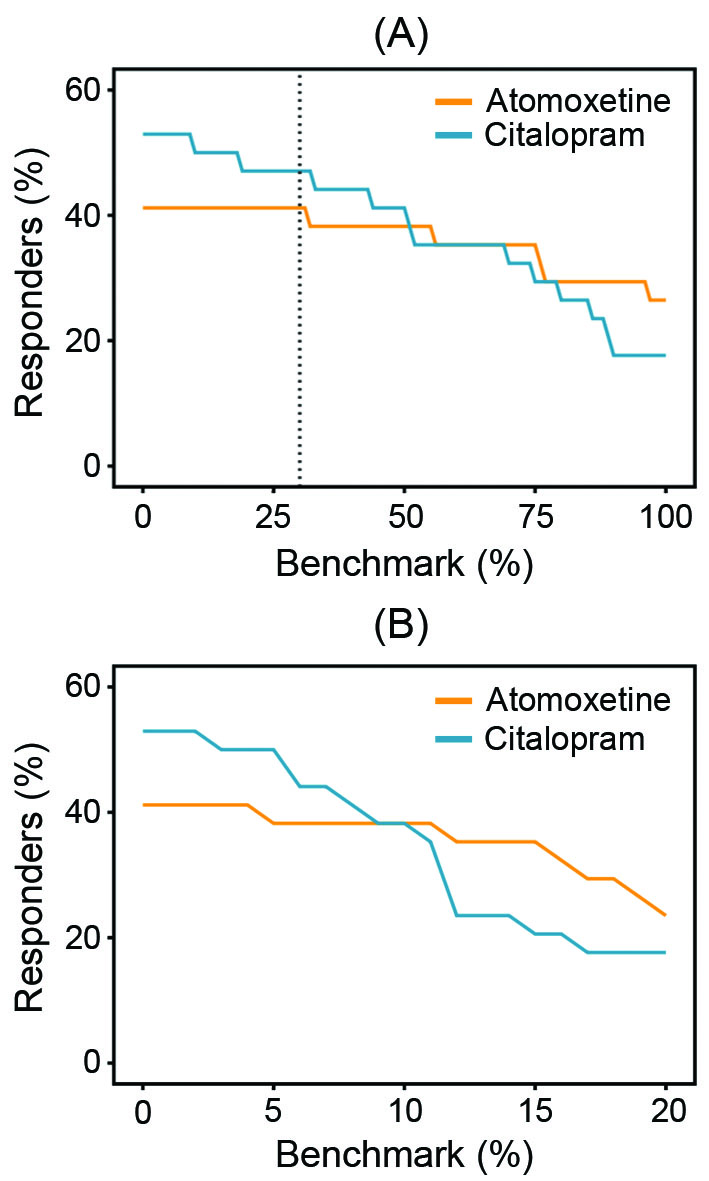


Permutation test

Figure S2: An illustration of the permutation approach, using the clinical model for atomoxetine response, against the principal benchmark of 30% behavioural improvement. The grey line indicates the distribution of cross-validation accuracy generated from 5000 randomizations. The black vertical line indicates the cross-validation accuracy of the real model. The red area shows the rate of random models that had cross-validation accuracy larger than the real model.


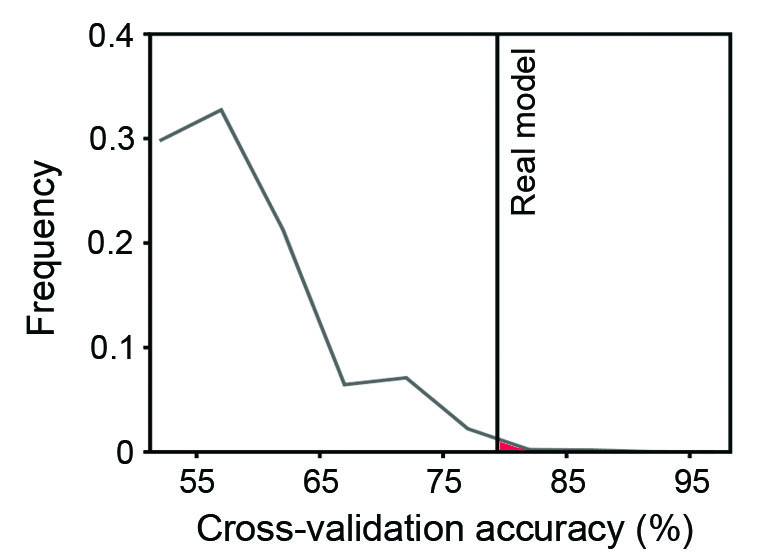


Behavioral results of the stop-signal task

Table S1: Mean behavioral measures (standard deviations) in the stop-signal task.

| **Measures** | **Control** | **PD-placebo** | **PD-atomoxetine** | **PD-citalopram** |
| --- | --- | --- | --- | --- |
| SSRT (ms) | 159 (46) | 194 (69) | 201 (70) | 198 (72) |
| Go reaction time (ms) | 509 (125) | 592 (110) | 590 (113) | 599 (113) |
| Go error rate (%) | 1.4 (1.5) | 3.3 (3.3) | 3.6 (3.6) | 3.7 (4.0) |

Table S2: The effect of each drug at the group level was tested with a repeated-measures ANCOVA covarying individual differences in the clinical and demographic variables as listed.

| **Main effect/interaction** | **Atomoxetine** | **Citalopram** |
| --- | --- | --- |
| Drug | ns | *p*<0.05 |
| Drug * Disease severity | ns | *p*<0.05 |
| Drug * Cognitive status | ns | ns |
| Drug * Levodopa equivalent dose | ns | ns |
| Drug * Plasma drug concentration | ns | ns |
| Drug * Age | ns | ns |

ns, not significant, *p*>0.07.

Results of additional NoGo trials

The stop-signal task had 40 additional trials with the stop-signal delay set to 0ms, equivalent to NoGo trials. We analyzed the behavioral performance and brain activity of these trials separately because they were not the focus of this study.

*Behavioral results*

The error rate of NoGo trials was 2.3% (standard deviation 5.7%) in control subjects. In patients with Parkinson’s disease, the error rate was 4.5% (4.0%) under placebo, 4.5% (7.2%) under atomoxetine, and 6.3% (10.8%) under citalopram. We examined group difference on NoGo error rate (PD-placebo *versus* control) using a two-sample two-tailed *t*-test. This test confirmed a trend towards an adverse effect of Parkinson’s disease (*t*=1.90, *p*=0.06). We then examined the effect of each drug separately (atomoxetine/citalopram *versus* placebo) using the repeated-measures ANOVA, controlling for age, disease severity (UPDRS-III motor subscale), cognitive status (MMSE), levodopa equivalent dose, and plasma drug concentration. There was a statistically significant interaction between citalopram and disease severity (*F*=6.19, *p*<0.05), indicating that citalopram reduced the error rate in patients with more advanced disease (higher UPDRS-III motor score; Figure S3).

Figure S3: Citalopram reduced the error rate of NoGo trials in patients with more advanced disease (higher UPDRS-III motor score). ∆Error indicates the change in error rate after citalopram *versus* placebo.


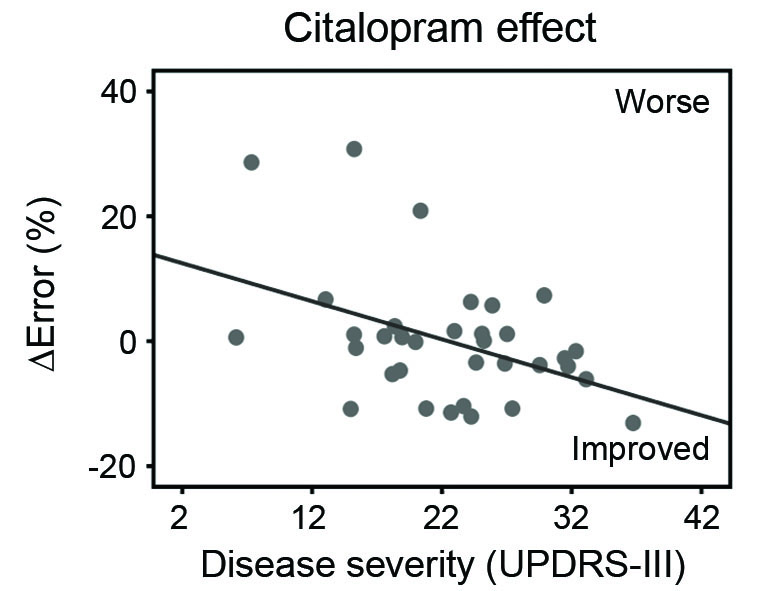


*fMRI results*

In the fMRI analysis, the NoGo trials were modeled separately in each subject’s general linear model. We computed the “NoGo>Go” contrast in each group (one-sample *t*-tests, voxel level *p*<0.001, cluster level *p*<0.05 family-wise-error-corrected for multiple comparisons) and examined group differences on NoGo-related brain activation (PD-placebo *versus* control; two-sample *t*-test, voxel level *p*<0.001, cluster level *p*<0.05 family-wise-error-corrected for multiple comparisons).

Control subjects showed greater activations in the right inferior frontal gyrus (peak in MNI coordinates [40, 8, 26], *t*=10.04, 1608 voxels) and pre-supplementary motor area ([16, 10, 64], *t*=5.77, 345 voxels) for NoGo *versus* Go trials (Figure S4A). Although the patients on placebo did not show significant activations in either SMA or inferior frontal gyrus, the group difference was not significant (noting a trend in the right inferior frontal gyrus: [34, 20, -6], *t*=4.46, 66 voxels, voxel-level *p*<0.001 uncorrected, cluster-level *p*<0.05 uncorrected).

We also examined the effect of each drug on the right inferior frontal gyrus, pre-supplementary motor area, caudate nucleus and putamen separately using the region-of-interest analysis similar to that for the stop-signal trials presented in the main text (Figure S4B). The repeat-measures ANOVA had drug as a within-subject factor and controlled for age, disease severity, cognitive status, levodopa equivalent dose, and plasma drug concentration. Atomoxetine, but not citalopram, enhanced the activation of the right inferior frontal gyrus for NoGo *versus* Go trials in patients with a lower drug concentration (*F*=7.85, *p*<0.01). But the increase in the activation of the right inferior frontal gyrus was not significant at the level of the whole group, ignoring individual differences. No significant drug effect was observed in the pre-supplementary motor area, caudate nucleus or putamen.

Figure S4: fMRI results of the NoGo trials. (A) Control subjects showed greater activations in the right inferior frontal gyrus and pre-supplementary motor area for NoGo *versus* Go trials. The group difference was not significant between control subjects and patients with Parkinson’s disease under placebo (PD-PLA). (B) Atomoxetine enhanced the activation of the right inferior frontal gyrus in patients with a lower plasma drug concentration.


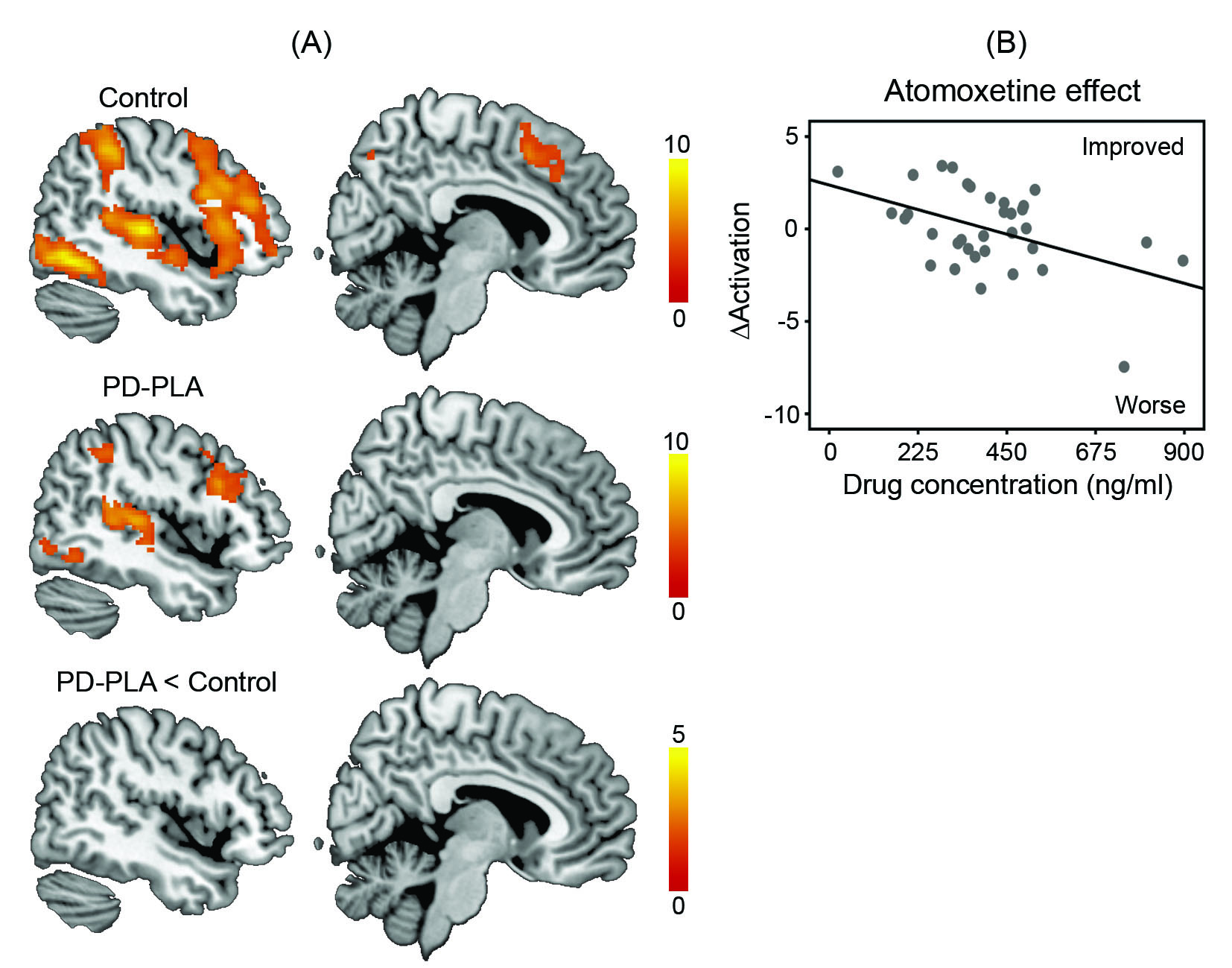


Whole-brain analysis of the two separate cohorts

For both cohorts, control subjects showed greater stop-related activation in the right inferior frontal gyrus. The stop-related activation was significantly weaker in patients with Parkinson’s disease after placebo (PD-PLA; disease effect, p<0.05 corrected). Slices are chosen to optimize visual illustration. Coordinates are in the MNI space.

Figure S5 Cohort 1

Figure S6 Cohort 2 (newly recruited and tested following the same research protocol)


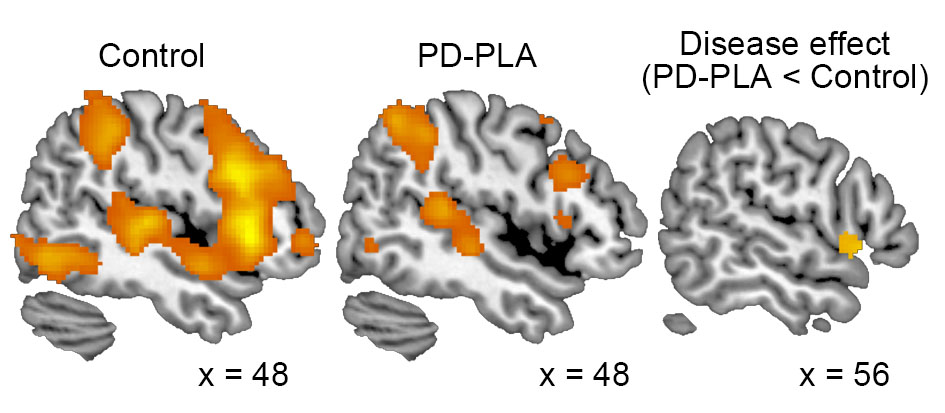


Effect of each drug on other brain regions

Regions such as pre-supplementary motor areas (pre-SMA) and striatum are commonly cited in the literature. We also analyzed the effect of each drug on the pre-SMA, caudate nuclus and putamen.

The pre-SMA region was defined as the intersection of the anatomical definition (Automated Anatomical Labelling) and “successful-stop>Go” contrast in control subjects. The regions of the caudate nucleus and putamen were anatomically defined because they were not significantly activated in our cohort. As for the right inferior frontal gyrus, we extracted parameter estimates for “successful-stop>Go” per subject per session and used the repeated-measures ANCOVA covarying individual differences in age, disease severity, cognitive status, levodopa equivalent dose, and plasma drug concentration.

The ANCOVA showed that the drugs increased the activation of the caudate nuclei bilaterally in older patients (left: atomoxetine, *F*=11.92, *p*<0.005; citalopram: *F*=5.86, *p*<0.05; right: atomoxetine, *F*=7.41, *p*=0.01). No drug effect was observed on the pre-SMA or putamen.

Sensitivity and specificity of the optimal models

We assessed sensitivity and specificity of the optimal models against the main benchmark (30% behavioral improvement) in the form of receiver operating characteristic (ROC) curves (Figure S7).

The ROC curve shows the true positive rate (i.e. the fraction of true positives out of the total actual positives; index of sensitivity) as a function of the false positive rate (i.e. the fraction of false positives out of the total actual negatives; index of 1-specificity). To generate the ROC curve, a pair of true/false positive rates was calculated and ranked after each validation test. The diagonal line is the line of no-discrimination (random). The closer the ROC curve is to the upper left corner (high sensitivity, high specificity), the better a model is.

Figure S7 ROC curves for (A) atomoxetine and (B) citalopram models against the main benchmark. The curves of clinical and mechanistic models are presented in the same graph for simplicity. The choice between the model types is not based on their differences in accuracy, but rather whether one is trying to make a clinical prediction or understand *post hoc* the drug effect in terms of brain activity.

**
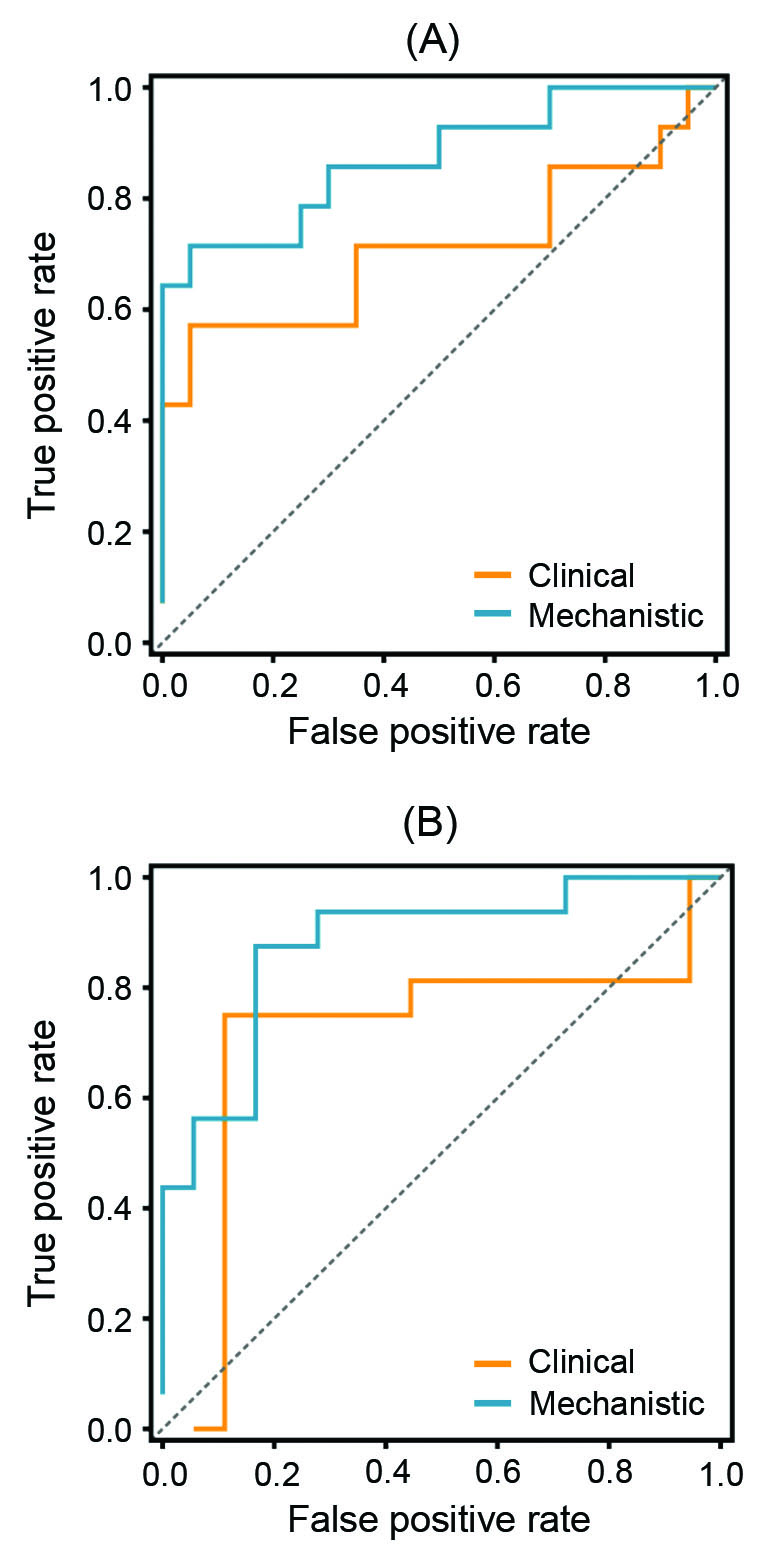
**

Models optimized against alternative benchmarks

Table S3: Optimal clinical predictive models for alternative benchmarks defined as the percentage of the magnitude of Parkinson’s deficit in SSRT.

| **Benchmark** | | **Optimal features** | **(*C*, *γ*)** | **Accuracy** |
| --- | --- | --- | --- | --- |
| *Atomoxetine* | | | | |
| 10% | L mean diffusivity, levodopa equivalent dose, R fractional anisotropy, L fractional anisotropy | | (2^10^, 2^3^) | 76.5% |
| 20% | L mean diffusivity, levodopa equivalent dose, R fractional anisotropy, L fractional anisotropy | | (2^10^, 2^3^) | 76.5% |
| 40% | R fractional anisotropy, levodopa equivalent dose, L fractional anisotropy | | (2^4^, 2^3.5^) | 79.4% |
| 50% | R fractional anisotropy, levodopa equivalent dose, L fractional anisotropy | | (2^4^, 2^3.5^) | 79.4% |
| *Citalopram* | | | | |
| 10% | MMSE, levodopa equivalent dose, UPDRS | | (2^10^, 2^-1^) | 76.5% |
| 20% | R fractional anisotropy, age, R mean diffusivity, MMSE | | (2^10^, 2^0.5^) | 79.4% |
| 40% | Age, R mean diffusivity | | (2^7.5^, 1) | 76.5% |
| 50% | R mean diffusivity, L mean diffusivity, age | | (2^2^, 2^4^) | 73.5% |

Values of fractional anisotropy and mean diffusivity were extracted from the anterior internal capsule. L, left; R, right; UPDRS, Unified Parkinson's Disease Rating Scale (III motor subscale); MMSE, mini mental state examination.

Table S4: Optimal mechanistic models for alternative benchmarks defined as the percentage of the magnitude of Parkinson’s deficit in SSRT.

| **Benchmark** | | **Optimal features** | **(*C*, *γ*)** | **Accuracy** |
| --- | --- | --- | --- | --- |
| *Atomoxetine* | | | | |
| 10% | R caudate nucleus, L caudate nucleus, R pre-SMA | | (1, 2^7^) | 85.3% |
| 20% | R caudate nucleus, L caudate nucleus, R pre-SMA | | (1, 2^7^) | 85.3% |
| 40% | L caudate nucleus, R putamen, R pre-SMA | | (2^8^, 2^2.5^) | 85.3% |
| 50% | L caudate nucleus, R putamen, R pre-SMA | | (2^8^, 2^2.5^) | 85.3% |
| *Citalopram* | | | | |
| 10% | R caudate nucleus, R IFG, R putamen, L putamen | | (2^6.5^, 2^-0.5^) | 82.4% |
| 20% | L caudate nucleus, R putamen, R pre-SMA | | (2^6^, 2^2^) | 85.3% |
| 40% | L caudate nucleus | | (2^6.5^, 2^7.5^) | 88.2% |
| 50% | L caudate nucleus | | (2^6.5^, 2^7.5^) | 85.3% |

L, left; R, right; pre-SMA, pre-supplementary motor area; IFG, inferior frontal gyrus.

Table S5: Optimal models for atomoxetine response, against a range of benchmarks defined as the percentage of individual SSRT baseline

| **Benchmark** | **Optimal features** | **(*C*, *γ*)** | **Accuracy** |
| --- | --- | --- | --- |
| *Clinical predictive models* | | | |
| 3% | R fractional anisotropy, plasma drug concentration, levodopa equivalent dose | (2^3.5^, 2^2^) | 79.4% |
| 5% | R fractional anisotropy, plasma drug concentration | (2^4.5^, 2^5^) | 76.5% |
| 8% | R fractional anisotropy, plasma drug concentration | (2^4.5^, 2^5^) | 82.4% |
| 10% | R fractional anisotropy, plasma drug concentration | (2^4.5^, 2^5^) | 82.4% |
| *Mechanistic models* | | | |
| 3% | R caudate nucleus, L caudate nucleus, R pre-SMA | (1, 2^7^) | 85.3% |
| 5% | L caudate nucleus, R putamen | (2^10^, 2^0.5^) | 79.4% |
| 8% | L caudate nucleus, R putamen, R pre-SMA | (2^8^, 2^2.5^) | 85.3% |
| 10% | L caudate nucleus, R putamen, R pre-SMA | (2^8^, 2^2.5^) | 85.3% |

Values of fractional anisotropy were extracted from the anterior internal capsule. pre-SMA, pre-supplementary motor area; L, left; R, right.

Table S6: Optimal models for citalopram response, against a range of benchmarks defined as the percentage of individual SSRT baseline

| **Benchmark** | **Optimal features** | **(*C*, *γ*)** | **Accuracy** |
| --- | --- | --- | --- |
| *Clinical predictive models* | | | |
| 3% | Plasma drug concentration, UPDRS, levodopa equivalent dose | (2^5^, 2^2.5^) | 76.5% |
| 5% | MMSE, levodopa equivalent dose, UPDRS | (2^10^, 2^-1^) | 76.5% |
| 8% | levodopa equivalent dose, MMSE | (2^10^, 2^-0.5^) | 73.5% |
| 10% | L mean diffusivity | (1, 2^9^) | 73.5% |
| *Mechanistic models* | | | |
| 3% | L caudate nucleus | (2^3^, 2^7.5^) | 79.4% |
| 5% | L caudate nucleus | (2^3^, 2^7.5^) | 82.4% |
| 8% | L caudate nucleus | (2^6.5^, 2^7.5^) | 85.3% |
| 10% | L caudate nucleus | (2^5.5^, 2^7.5^) | 85.3% |

Values of mean diffusivity were extracted from the anterior internal capsule. UPDRS, Unified Parkinson's Disease Rating Scale (III-motor subscale); MMSE, mini mental state examination; IFG, inferior frontal gyrus; L, left; R, right.

Models optimized with different feature inputs

We also optimized the models with different feature inputs, against a range of benchmarks defined as the percentage of Parkinson’s deficit in SSRT. The additional analysis showed the relative importance of each feature in a given model (e.g. whether the prediction accuracy changed dramatically when a particular feature was added into or removed from the model).

Table S7 shows the models with all available measures, including the demographic, clinical, and structural and functional imaging measures. Note that the availability of functional imaging measures (drug-induced changes of brain activation) as feature inputs may lead to the exclusion of demographic, clinical and structural imaging measures as not being sufficiently contributory to the optimal models, as in the case of atomoxetine.

The clinical predictive models were also optimized using the measure of plasma drug concentration (Table S8), or without using any imaging measures (i.e. only demographic and clinical measures, Table S9). These models demonstrated that the powerful technique we used is applicable to different situations (e.g. when a particular measure is unavailable).

Table S7: Optimal mixed models with all measures as feature inputs, against a range of benchmarks defined as the percentage of Parkinson’s deficit in SSRT.

| **Benchmark** | **Optimal features** | **(*C*, *γ*)** | **Accuracy** |
| --- | --- | --- | --- |
| *Atomoxetine* | | | |
| 10% | R caudate nucleus, L caudate nucleus, R pre-SMA | (1, 2^7^) | 85.3% |
| 20% | R caudate nucleus, L caudate nucleus, R pre-SMA | (1, 2^7^) | 85.3% |
| 30% | R caudate nucleus, L caudate nucleus, R pre-SMA | (1, 2^7^) | 85.3% |
| 40% | L caudate nucleus, R putamen, R pre-SMA | (2^8^, 2^2.5^) | 85.3% |
| 50% | L caudate nucleus, R putamen, R pre-SMA | (2^8^, 2^2.5^) | 85.3% |
| *Citalopram* | | | |
| 10% | R IFG, R putamen, UPDRS, R mean diffusivity | (2^10^, 2^1.5^) | 88.2% |
| 20% | UPDRS, R putamen, R mean diffusivity, R IFG | (2^10^, 2^0.5^) | 88.2% |
| 30% | UPDRS, R putamen, R mean diffusivity, R IFG | (2^10^, 2^0.5^) | 88.2% |
| 40% | L caudate nucleus | (2^6.5^, 2^7.5^) | 88.2% |
| 50% | L caudate nucleus | (2^6.5^, 2^7.5^) | 85.3% |

Values of mean diffusivity were extracted from the anterior internal capsule. L, left; R, right; pre-SMA, pre-supplementary motor area; IFG, inferior frontal gyrus; UPDRS, Unified Parkinson's Disease Rating Scale (III motor subscale).

Table S8: Optimal clinical models with plasma drug level, against a range of benchmarks defined as the percentage of Parkinson’s deficit in SSRT.

| **Benchmark** | | **Optimal features** | **(*C*, *γ*)** | **Accuracy** |
| --- | --- | --- | --- | --- |
| *Atomoxetine* | | | | |
| 10% | R fractional anisotropy, plasma drug concentration, levodopa equivalent dose | | (2^3.5^, 2^2^) | 79.4% |
| 20% | R fractional anisotropy, plasma drug concentration, levodopa equivalent dose | | (2^3.5^, 2^2^) | 79.4% |
| 30% | R fractional anisotropy, plasma drug concentration, levodopa equivalent dose | | (2^3.5^, 2^2^) | 79.4% |
| 40% | R fractional anisotropy, plasma drug concentration | | (2^4.5^, 2^5^) | 82.4% |
| 50% | R fractional anisotropy, plasma drug concentration | | (2^4.5^, 2^5^) | 82.4% |
| *Citalopram* | | | | |
| 10% | MMSE, levodopa equivalent dose, UPDRS | | (2^10^, 2^-1^) | 76.5% |
| 20% | R fractional anisotropy, age, R mean diffusivity, MMSE | | (2^10^, 2^0.5^) | 79.4% |
| 30% | R fractional anisotropy, age, R mean diffusivity, MMSE | | (2^10^, 2^0.5^) | 79.4% |
| 40% | Age, R mean diffusivity | | (2^7.5^, 1) | 76.5% |
| 50% | R mean diffusivity, L mean diffusivity, age | | (2^2^, 2^4^) | 73.5% |

Values of fractional anisotropy and mean diffusivity were extracted from the anterior internal capsule. L, left; R, right; UPDRS, Unified Parkinson's Disease Rating Scale (III motor subscale); MMSE, mini mental state examination.

Table S9: Optimal clinical predictive models, without using brain imaging measures (only demographic and clinical measures).

| **Benchmark** | **Optimal features** | **(*C*, *γ*)** | **Accuracy** |
| --- | --- | --- | --- |
| *Atomoxetine* | | | |
| 10% | Plasma drug concentration | (2^9^, 2^6.5^) | 73.5% |
| 20% | Plasma drug concentration | (2^9^, 2^6.5^) | 73.5% |
| 30% | Plasma drug concentration | (2^9^, 2^6.5^) | 73.5% |
| 40% | Plasma drug concentration, levodopa equivalent dose | (2^7.5^, 2^1.5^) | 79.4% |
| 50% | Plasma drug concentration, levodopa equivalent dose | (2^7.5^, 2^1.5^) | 79.4% |
| *Citalopram* | | | |
| 10% | MMSE, levodopa equivalent dose, UPDRS | (2^10^, 2^-1^) | 76.5% |
| 20% | levodopa equivalent dose, MMSE | (2^9.5^, 2^-0.5^) | 76.5% |
| 30% | levodopa equivalent dose, MMSE | (2^9.5^, 2^-0.5^) | 76.5% |
| 40% | levodopa equivalent dose, MMSE | (2^10^, 2^-1^) | 76.5% |
| 50% | levodopa equivalent dose, MMSE | (2^10^, 2^-0.5^) | 70.6% |

UPDRS, Unified Parkinson's Disease Rating Scale (III motor subscale); MMSE, mini mental state examination.

**References**

Ye Z, Altena E, Nombela C, Housden C, Maxwell H, Rittman T, Huddleston C, Rae CL, Regenthal R, Sahakian BJ and others. (2014): Selective serotonin reuptake inhibition modulates response inhibition in Parkinson’s disease. Brain 137(Pt4):1145-55.

Ye Z, Altena E, Nombela C, Housden C, Maxwell H, Rittman T, Huddleston C, Rae CL, Regenthal R, Sahakian BJ and others. (2015): Improving response inhibition in Parkinson's disease with Atomoxetine. Biological Psychiatry 77(8):740-8.
